# Comparative analysis of newly graduated nurse support through periods of turmoil: lessons learnt for building a future workforce for uncertain times

**DOI:** 10.1186/s12912-024-02460-4

**Published:** 2024-10-30

**Authors:** Casey Riches, Rachael Pitt, Scott Tyler, Megan Wise, Philip Watman, Amanda Henderson

**Affiliations:** 1 Princess Alexandra Hospital, 199 Ipswich Road, Woolloongabba, Brisbane, QLD 4102 Australia; 2 School of Nursing, Midwifery, and Social Sciences, Central Queensland University, 160 Ann Street, Brisbane, QLD 4102 Australia; 3 Primary Care Partnerships Unit, Metro South Health, Eight Mile Plains, PO Box 4195, Brisbane, QLD 4113 Australia

**Keywords:** Stability, Instability, Graduate nurse, Support programs, Nursing education, Nursing retention, Nursing workforce

## Abstract

**Aim:**

To compare newly graduated nurse retention and employment experiences across two distinct graduate nurse programs through periods of health care delivery stability and instability.

**Background:**

A global nursing workforce shortage, coupled with increasing demands on health services, requires a specific focus on building capability and improved retention of nurses. The graduate nurse cohort is a critical supply source that potentially can be harnessed if their needs are better understood.

**Design:**

A longitudinal (2015–2023) cross-sectional design was used to examine retention and experiences of newly graduated nurses from two (‘traditional, unit-based’ versus ‘mobile’) supported programs.

**Methods:**

Retention rates were obtained through analysis of employment databases. Descriptive data on impressions and experiences was collected at two time points via a short series of questions contained in a survey, from graduate nurses who remained in employment.

**Results:**

Retention rates for graduate nurses were high (85%) during periods of stability, but even higher for the ‘mobile’ graduate nurse program. Both programs were adversely affected by instability however, the impact was substantial (around 50%) for the peak period of instability (2020) in the ‘mobile’ program. Survey data indicated that during the period of instability graduate nurses in traditional, unit-based programs had a more positive experience when compared with graduate nurses participating in the ‘mobile’ program; This contrasts with the period of stability, where graduate nurses in the ‘mobile’ program indicated more positive responses.

**Discussion:**

Findings re-emphasise the importance of supportive structures for newly graduated nurses as effective in retaining these staff. However, for the first time, evidence is provided that ‘mobility’, diversity, and difference can be viewed positively by graduate nurses. Furthermore, it provides improved understanding about mechanisms, support and boundaries, all interrelating factors, in ‘stretching’ capacity of graduate nurses.

**Conclusion:**

Longitudinal analysis of graduate nurses, participating in two diverse programs, through periods of stability and instability offers insights into their challenges and outcomes. These insights can directly inform targeted strategies for inclusion in supported programs that lead to improved graduate nurse retention and contribute to building an agile nursing workforce.

**Supplementary Information:**

The online version contains supplementary material available at 10.1186/s12912-024-02460-4.

## Introduction

Demands on the provision of adequate, quality healthcare services continue to pressure nursing workforces globally. Academic literature reports how tailored graduate nurse programs that include assistance with professional and social adaption can address the emerging nursing workforce shortage to benefit health service organisations and newly graduated nurses [1–3]. Contemporary approaches to the structure and delivery of graduate nurse programs have been advocated to better meet organisational workforce requirements and support retention of newly graduated nurses [1, 4]. To aid this purpose, this study compared graduate nurse retention and employment experiences, specifically organisational development processes, across two distinct graduate nurse programs (unit-based and ‘mobile’) over seven years, inclusive of the pandemic period when health systems were in crisis. This study, in the specific context of a major tertiary hospital in South-east Queensland, Australia, sought to explore the benefits to and demonstrate the elements integral to building an agile nursing workforce to address challenges faced in the changing landscape of healthcare, now and into the future.

## Background

Establishment of an agile, capable nursing workforce is prudent given the current and future demands on health care delivery, not the least being, the unpredictability of global health urgencies. Supportive and responsive workplace programs for nurses when they commence employment better position health services to meet patient needs now and into the future; especially programs that build agility [3]. Nurses who feel confident and capable about their care provision are more likely to derive satisfaction and remain in employment [1, 2, 4, 5]. Newly graduated nurses are predicted to comprise a significant proportion of the nursing workforce in coming years, as the current cohort of nursing staff move toward retirement. A global nursing workforce shortage, coupled with increasing demands on health services resulting from the global pandemic and an ageing population, requires a specific focus on building capability and improved retention. The challenge is to better engage and support newly graduated nurses to create a capable, responsive, sustainable workforce.

### Global nursing shortage and attrition

The global shortage of nurses internationally was estimated to be 5.9 million prior to the pandemic [6]. Difficulties with nurse retention are proposed following the pandemic due to numerous person-specific reasons including chronic disease, burnout, and early retirement, which are exacerbated by workplace issues, such as increased staff-patient ratios, an ageing population and greater patient acuity; all of which intensify existing nurse workforce shortages [6]. Nurse retention is a known worldwide issue, and it is well-documented that many nurses are planning to leave the profession [7].

Internationally, graduate nurses are being recruited in significant numbers to replace nurses leaving the profession. Australia is well positioned to replenish their workforce through graduate nurse recruitment. Data from the Organisation for Economic Co-operation and Development iLibrary [8] from 2021 reports Australia, with 116 new graduate nurses per 100,000 population has the second greatest proportion of nurses per capita globally. Potentially, Australia may have an oversupply of newly graduated nurses readily able to reinstate the diminishing workforce [9]. Targeting this newly graduated nurse workforce is appropriate to address a looming void. It is acknowledged that millennials, identified as people born between 1981 and 1999, constitute a significant proportion of this graduate cohort, and value career purpose and direction, strong leadership, and advancement opportunities as priorities in their work [2, 10].

Millennials are more likely than previous generations to leave their role when organisational and personal values do not align [2]. Arguably, opportunities for diverse and varied experiences were forthcoming during the crisis of the pandemic as health care priorities needed to pivot quickly; primary care shifted from containment and tracking, to vaccination, and focused responses to management of COVID-19. Potentially, the diversity of work experiences on offer could well suit the emerging millennial workforce. The pandemic was a turbulent time. It was accompanied by unprecedented pressures and stressors for students completing their studies and entering the workforce. Research consistently reports high stress levels for students and graduates [11, 12], and retaining the workforce was a significant challenge.

### Retention and graduates

The literature continues to surmise that when known challenges for newly graduated nurses are addressed, they effectively manage situations, they are more satisfied, and they remain in the workforce [1, 4]. These challenges can be addressed through specifically designed and adequately resourced workplace programs that advance competence and assist with preparedness; such programs identify ‘gaps in practice’, provide timely emotional support to foster adjustment to the work environment, and develop confidence through constructive guidance of problem-solving thereby advancing professional and social adaption to the workplace [11, 13–15]. Rapid reviews can inform contemporary interpretation of elements that cater for workplace needs. We undertook a rapid review that used the key terms: graduate, novice, early career nurse, retention, turnover, attrition, to derive contemporary evidence regarding retention, workplace opportunities and provisions by the health facilities. The period 2014–2022 was selected as prior to this, new graduate nurse experiences had already been positively reported when programs addressed known learning and support needs [16]. It was anticipated the period 2014–2022 would potentially include novel information related to working through turbulent times.

The rapid review located just seven papers focused specifically on the relationship between graduate nurse retention with the elements of graduate programs. All papers had MMAT scores of 60% or over. Analysis of these seven papers re-affirmed the essential components of graduate nurse programs; namely, adequate learning and support that addressed graduate nurse challenges of assisting assimilation into their units through building capability and managing emotional distress of graduate nurses as most salient for retention (refer Table 1). Two of these studies measured actual turnover [17, 18]; the remainder studies measured turnover intention.

**Table 1:** Details of studies in rapid review

Author, (year)	Theme	Method/design	Participant demographic and study sample	Factors influencing retention	Quality Score
Cao et al. (2021) [19]	Transition shock, workplace culture	Quantitative/ cross sectional descriptive study	361 Chinese newly graduated nurses with < 1 work experience holding a Chinese nursing license (N.B. not specifically a Bachelor degree)	Resilience (nurse managers could provide resilience training). Work environment (nurse managers could develop a supportive workplace climate). Transition shock (emotional burden).	MMAT 100%
Dwyer et al. (2019) [20]	Structural empowerment, workplace culture, transition shock	Quantitative/ cross sectional exploratory design/descriptive study	136 newly licensed graduate nurses working in their first job (between 6 months to 3 years)	Workplace environment, workplace relationships and intrapersonal characteristics influence transitional outcomes.	MMAT 60%
Favaro et al. (2021) [21]	Structural empowerment	Quantitative/ secondary analysis – non-experimental, descriptive correlational study	1008 male or female graduate nurses with 12–24 months experience, registered with one of the 10 provincial registered nursing bodies in Canada	Structural empowerment may be used to reduce the prevalence of bullying and consequently reduce job turnover intention.	MMAT 100%
Han et al. (2019) [17]	Workplace culture	Mixed methods/ descriptive and prospective longitudinal study	464 newly graduated nurses who had no prior nursing work experience	Hospitals to implement organisational and educational initiatives to encourage healthy lifestyles.	MMAT 80%
Kaihlanen et al. (2020) [22]	Transition shock	Quantitative/ cross sectional survey study	712 registered nurses graduated within the past two years	Transition from clinical practicum to nursing role – namely emotional (psychological distress) and socio-developmental (role conflict and ambiguity) domains.	MMAT 80%
Koneri et al. (2021) [18]	Workplace culture	Quantitative/ nonrandomised trial	125 newly graduated nurses who completed a one-year residency program	Touchpoints that aligned with organisational values improved retention of new graduates (care cards, newsletters featuring graduate nurses, coffee & chat engagements, interprofessional support).	MMAT 60%
Tyndall et al. (2019) [10]	Structural empowerment	Mixed methods/ nonrandomised study	9883 graduate nurses across 30 healthcare systems	Job embeddedness (the more a nurse identifies with the employing organization, the higher the level of job embeddedness and satisfaction).	MMAT 60%

Findings from this contemporary rapid review underscore that retention of graduate nurses is associated with coping with limited preparedness, often referred to as ‘transition shock’ and facilitating assimilation into the workplace [19–22]. An umbrella review that critically synthesises newly graduated nurses’ experiences summarises these into three overlapping themes, namely, ‘feeling a lack of competency’, ‘sense of emotional distress’, and ‘in need of support’ [23]. Professional and social adaptation are key to effective management of these experiences [1, 4, 24]. Shock is defined as unexpected and an unpleasant feeling. The themes summarised in the umbrella review suggest ‘shock’ for the graduate nurse related to limited help in adaptation. It is imperative, therefore, to investigate organisational and education supports that address these needs. Currently there are many tailored programs to assist adaptation. Structured to provide emotional support, individualised learning and build capability; these are afforded a range of names including, fellowships, graduate programs, transition to practice programs, graduate nurse residency [24].

During recent times, health services have been confronted with challenges, namely, work environments that need to be agile and responsive to effectively administer public health demands. Within this, graduate nurses are required to be agile, yet they also need clear process and structure to minimise the ‘unexpected’. Meeting competing demands of emotional well-being of newcomers in rapidly changing environments and work requirements is imperative to sustain a dwindling workforce. Effective engagement through tailored support programs is essential as benefits are twofold; to ease the financial burden of excessive staff turnover, and more importantly, to improve staff continuity and capability thereby optimising patient safety. While needs for assistance with professional, social and organisational adaptation are known, the best organisational approaches to meet graduate nurse needs are not clear [25]. Recent reviews of graduate nurse success advocates appropriate tailoring of support for comprehensive development of graduates, including social intelligence and adaptation, so as to meet personal needs and capacity to address organisational issues [1, 24]. Social intelligence, recognised as the ability to understand and manage personal relationships, and thereby directly assist social adaptation are of paramount importance during health care crisis. Newly graduated nurses are confronted with an extraordinary range of issues that warrant more than the standard capabilities usually assessed prior to gaining registration; social intelligence and accompanying constructive responsiveness significantly contribute to successfully addressing these issues.

### Alternative organisational and education support for newly graduated nurses

Crisis in health systems is not uncommon. Increasingly, the divergent and competing demands challenge staff and the systems that they are required to work in. These precedents have necessitated reviewing support for graduate nurses. About ten years ago the health facility in this study re-organised provision of support for graduate nurses: Further to the existing graduate program, a second, alternate pathway was created to build a versatile, responsive, and agile workforce. The new program sought to better prepare a workforce that manages rapidly changing circumstances crucial when circumstances are unpredictable. Traditionally, emergent staffing needs were substituted with a labile workforce referred to as ‘casual’, ‘bank’, ‘travel’, ‘pool’ or ‘float nurses’ [26–30]. Within this paper the substituted nurses are referred to as the ‘mobile’ workforce. These nurses are recognised as adept and flexible as patients and their place of work can vary enormously with each shift. However, limitations to transient labile workforces include lack of commitment and investment, as these nurses often do not feel a sense of belonging or achievement [31]; furthermore, staff supply may not be reliable, as transient workforces can choose when they want to work.

A guaranteed *readily available* graduate nurse workforce to respond to emergent needs in short time frames was deemed important for sustaining the workforce. A substantial team of newly graduated nurses was commenced through this alternate organisational program to ensure adequate workforce capacity in managing breadth and latitude of organisational imperatives. This program was purposefully designed to guide these graduate nurses to better master the ‘unexpected’. These graduate nurses are fostered to develop individual responsive and organisational acumen through one-on-one leadership development discussions, in contrast to specialised unit skill sets obtained through firmly defined processes and structures. Specifically, proactive engagement with newly graduated nurses and appropriate advocating during their shift, sought to focus development on building confidence and core skill competency attainment to assist adaptation. This involved steering graduate nurses’ development to succinctly verbalise scope, focus on setting priorities in care delivery and to seek opportunities. Additional to this, it is also important to broaden the graduate nurses’ conception and appreciation of nursing. With the World Health Organisation advocating for the address of sustainable development goals [32], the practice of protecting populations and optimising well-being is crucial to any nursing practice.

### Distinct graduate nurse programs to meet specific organisation needs

In the interests of developing a highly capable and responsive workforce that recognises and addresses the breadth of health care needs, a ‘novel’ graduate support program was conducted in parallel with the existing program: Program one, the existing unit-based graduate nurse program that invests detailed effort in the specialised nuances of dedicated medical and surgical units conducted over a 12 month period; and program two, a six-month program designed to build responsive and versatile nurses, where newly graduated nurses are placed in two primary rotations across different areas, for example, medicine and surgery. In program one, graduate nurses are employed in specific settings for one year, focused on building specialist capability consistent with clinical care provision in that designated unit. The facility has 24 specialist units, for example, orthopaedics, coronary care, oncology, respiratory, renal, head and neck surgery; whereby these graduate nurses are placed at one of these 24 units. In program two, following two rotations over a six-month period, nurses are placed in a variety of units based on staff vacancies and organisation needs each day. These nurses are coached to be receptive and responsive to accommodate change. The learning on offer to assist reaching the desired goals for both programs is commensurate, including orientation, monthly debriefing sessions, guided coaching at the bedside, focused study days, clinical instruction and workplace performance appraisal and feedback [33]. Each specialist clinical unit has a designated support team to coach and mentor. Similarly, ‘mobile’ newly graduated nurses have access to clinical facilitators from 0600 h until 2330 h, seven days per week, from a central resource, that they can request support in the unit they currently work. Opportunities to participate in study days are organised according to staffing demands of the organisation.

The management, processes, work location, and intention of these two programs are distinct; Therefore, a longitudinal analysis of their impact on recruitment, retention, and experience is warranted. Further to this, circumstances of COVID-19 during this longitudinal analysis afforded comparison of retention and experiences of nurses in challenging circumstances offering significant insights into expectations and reactions. During the pandemic, both programs required modifications. Newly graduated nurses employed in specialist units stayed in their home wards, yet much of their learning shifted from face-to-face to on-line modes, where possible (depending on current restrictions) face-to-face learning days incorporated physical distancing requirements. Alternatively, graduate nurses employed in the ‘mobile’ workforce were subjected to constant change as location and sphere of work was provided with short notice. Work setting and scope transitioned from testing centres, checking-in facility visitors, ‘mask fit’ testing, hotel quarantine, airport screening and vaccination centres in response to emerging and shifting public health policy requirements. Longitudinal scrutiny of graduate nurse retention alongside unit-based and ‘mobile’ employment experiences inclusive of turbulent times, namely, the pandemic, assists a nuanced understanding of graduate nurse experiences. The interest in this study is understanding of salient elements and circumstances of two discrete graduate support programs (unit-based and ‘mobile’); longitudinal analysis from prior to the pandemic, during, and immediately after affords significant insights.

## Methods

### Design

A longitudinal cross-section design survey of two distinct graduate nurse programs (unit-based and ‘mobile’).

### Setting

An acute tertiary care metropolitan health care facility in the southeast Queensland, Australia provided the setting for the study. The health care facility is a 1037 inpatient bed hospital with adult services offered in specialised surgical, medical, cancer and rehabilitation. Historically, the facility employs 100 to 170 newly graduated nurses annually. The exact number of new graduate nurses employed each year is dependent on nursing workforce staffing requirements.

### Participants

All graduate nurses employed at the facility were included in the retention data. Only the subset that remained at the time the survey was sent were able to complete the survey seeking feedback about their experiences.

*Retention rates* were derived for two graduate nurse cohorts at two time periods: (1) Nurses commencing at the facility in the four years preceding the pandemic (2015–2018) and still employed at the 21st week in 2019; and (2) Nurses commencing at the facility immediately after early waves and at the latter stages of the pandemic (2020–2022), and still employed at the 21st week in 2022.

Specifically for the purposes of comparison, retention rates were calculated as follows:

#### Period of stability

All graduate nurses who commenced employment at the tertiary facility from 1st January 2015 until mid-2018 [graduate nurse commencement is staggered over the first six months of the year] and who were still employed at the facility at 21st week in 2019 when the survey was distributed.; and.

#### Period of instability

All graduate nurses who commenced employment at the tertiary facility from 1st January 2020 until mid-2022 [graduate nurse commencement is staggered over the first six months of the year] and who were still employed at the facility at the 21st week in 2022 when the survey was distributed.

Graduate nurse *s’ experiences* were derived from a survey that was sent to all graduate nurses remaining in the facility at the time of the survey. The survey was sent at the two designated times recruitment data was analysed, namely, the 21st week in 2019, and the same week in 2022.

## Data collection

### Retention rates

Quantitative data of graduate nurses who commenced employment in 2015–2018, and then 2020–2022, and still remained at the metropolitan facility were located through employment databases.

### Graduate nurse experiences

Graduate nurses who commenced employment and were still employed at the facility were sent a short series of questions via a survey tool, developed specifically for this study, that sought information about their commencement date, graduate program, and experiences (see Appendix A for survey tool). Survey questions were constant across both time periods; except the addition of one question in the latter time period that asked about impact of COVID-19.

1. When did you complete your graduate year?
2. What Graduate Program did you complete [‘unit based’, or ‘mobile’]?
3. In what department did you commence your graduate year?
4. Do you still work in that unit? If not, where are you working now?
5. What is your Substantive Position?
6. How would you describe your graduate year in three words?

The additional one question asked was, “What was the impact of COVID-19 for you?”.

## Results

The number of newly graduated nurses that were recruited each year are detailed in Table 2 according to when they were recruited during a period of stability or instability, namely, graduates recruited from 2015 to 2018 and remained in the organisation at the 21st week of 2019 (period of stability); and then recruited following onset of the pandemic, 2020–2022, and remained employed at the latter, at the 21st week of 2022 (period of instability). The total number of graduates that commenced employment into the graduate program in the second period, during 2020 to 2022, was smaller than that of the first period of 2015 to 2018. This was due to an overall lower number of applicants that had applied to the facility for graduate positions post pandemic. This anomaly was outside the influence of both the higher education and health system. It was arguably due to a shift in the schooling system across the state of Queensland some fifteen years earlier that legislated an extra year of schooling prior to higher education, namely, a ‘pre-school year’. This year delay in pupils commencing school resulted in reduced numbers of students entering university (2018–2019), and therefore graduating from university three years later. Further to this, numbers of graduates were lower during the pandemic because completion of their studies were often delayed due to the restricted number of clinical placements.

### Retention data

The results for the period of stability 2015–2018 (refer Table 2) indicate that the number of graduate nurses who commenced each year in each program were greater for the ‘mobile’ workforce; especially, 2015 and 2016 when the ‘mobile’ graduate nurse program commenced as this was when the need for an agile, flexible workforce was anticipated. Of interest are high retention rates, 79–90% of graduate nurses across both programs commencing employment during 2015–2018 were remaining in the facility at the time of the survey. The retention rates were particularly high for the ‘mobile workforce’ programs; this difference equates to approximately 29 nursing staff, a significant saving for health facilities. A pilot study in Australia estimated staff turnover costs to be $A16,634 per nurse [34].

As this research was wanting to clearly distinguish between time periods of stability and instability, graduate nurses commencing during 2019 were not included: they commenced prior to the pandemic but then in the latter part of their transition (12 months to two years) would have been subjected to disruption therefore not clearly belonging in either period.

Retention rates for graduate nurses varied considerably during the pandemic, that is, the first wave during 2020, and then subsequent years when globally, health services were impacted (refer Table 2). The most severe impact was in 2020, only 63% retention, when world-wide public health was impacted, significantly affecting health facility services. Retention rates for both graduate nurse programs, unit-based (75%) and ‘mobile’ (50%), were adversely affected; however, the impact was greater for the ‘mobile’ program. This contrasted with the period of stability (prior to the pandemic).

Of interest, is that during the subsequent years, when there were still considerable disruptions to health care delivery to effectively manage COVID-19 patients and service provision, graduate retention improved (retention rate in 2021 was 80%; refer Table 2). Retention rates quickly resumed to rates observed during the period of stability, and this was across both graduate nurse programs. It is important to note, in 2021, following the immediate crisis only a small number of positions were offered to graduate nurses. Limited spaces available for graduate nurses may have contributed to reduced attrition.

**Table 2:** Number of new graduate nurses commencing employment 2015–2018 and 2020–2022

Year commenced	Total graduate nurses		Unit-based graduate nurses		Mobile graduate nurses	
	Commenced *n*	Remaining *n* (%)	Commenced *n*	Remaining *n* (%)	Commenced *n*	Remaining *n* (%)
2015	180	145 (81%)	74	61 (82%)	106	84 (79%)
2016	166	129 (78%)	81	56 (69%)	85	73 (86%)
2017	131	125 (96%)	56	51 (91%)	75	74 (99%)
2018	144	132 (85%)	82	65 (79%)	67	62 (92%)
**2015-2018 Totals**	**621**	**531 (85%)**	**293**	**233 (80%)**	**328**	**298 (91%)**
2020*	171	107 (63%)	87	65 (75%)	84	42 (50%)
2021	96	77 (80%)	52	40 (78%)	44	37 (85%)
2022	163	163 100%)	109	109 (100%)	54	54 (100%)
**2020-2022 Totals**	**430**	**347 (80%)**	**248**	**214 (86%)**	**182**	**133 (73%)**

*2019 omitted to clearly distinguish between time periods of ‘stability’ and ‘instability’

### Survey results

The survey sought to make sense of the retention data by seeking comments and differentiating them according to periods of stability and instability. The response rates for the survey distributed 2015–2018 was 176 out of 531 (31%), and during 2020–2022 was 93 out of 347 (27%). Approximately 30% of graduate nurses in the workforce at the time the survey was distributed, completed the survey. This response rate is typical of these populations.

#### Question six – “How would you describe your graduate year in three words?”

**Fig. 1:**
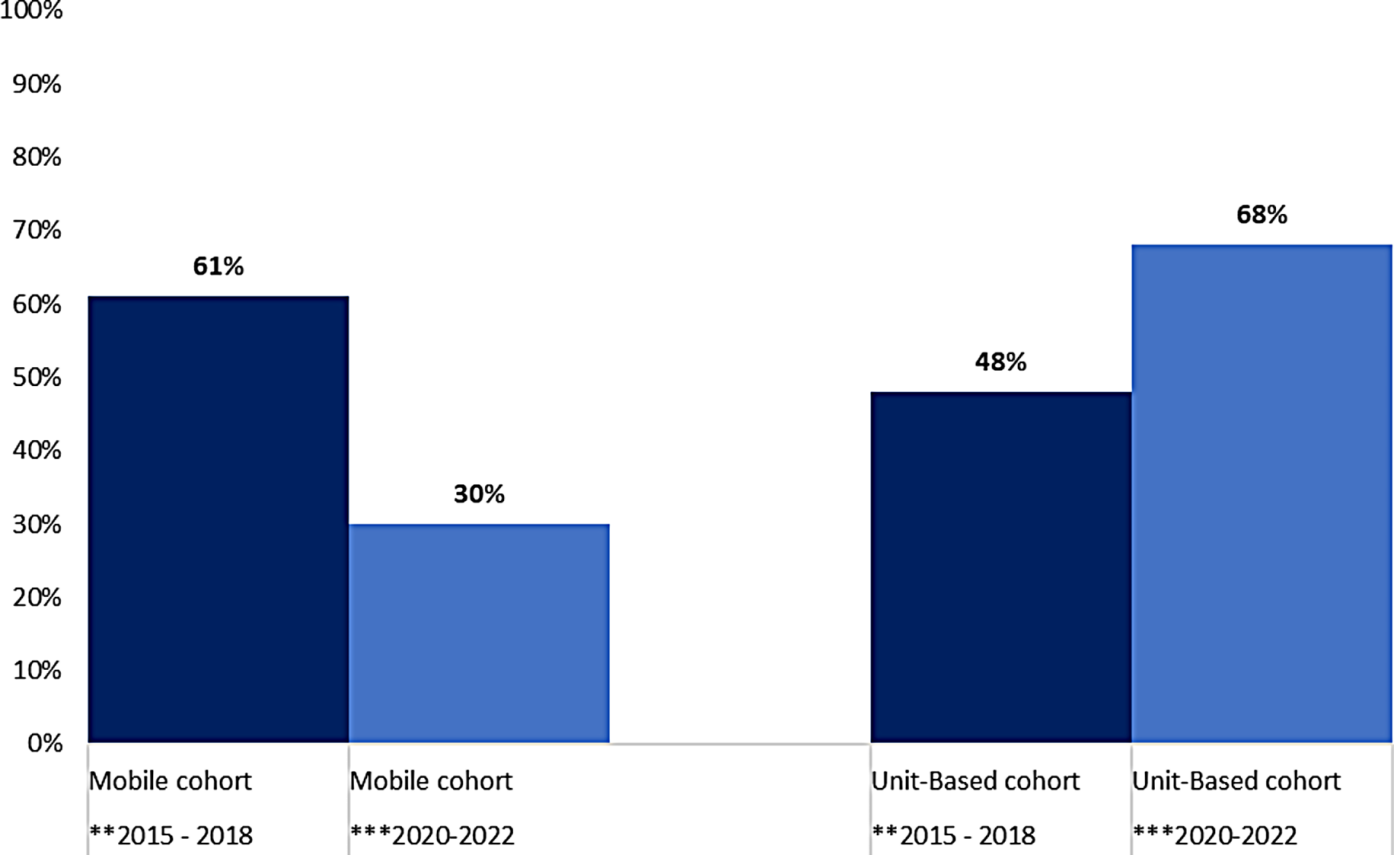
Percentage of words describing positive experiences by newly graduated nurses, between two distinct time periods. **period of stability (before COVID-19 pandemic). *** period of instability (during and immediately after the early waves of COVID-19 pandemic)

**Fig. 2:**
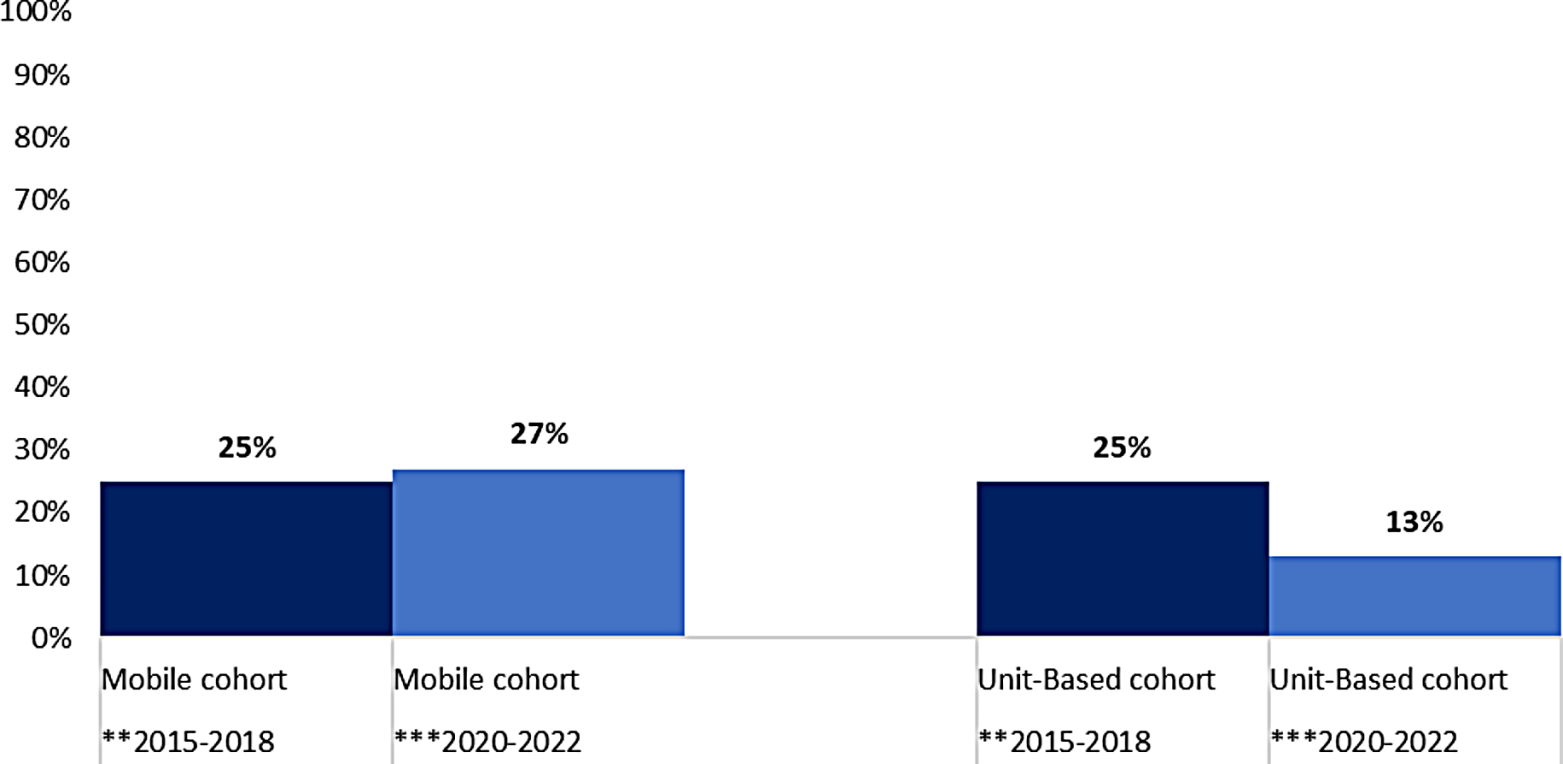
Percentage of words describing negative experiences by newly graduated nurses, between two distinct time periods. **period of stability (before COVID-19 pandemic). *** period of instability (during and immediately after the early waves of COVID-19 pandemic)

Question six of the survey that asked respondents to describe their graduate nurse experience in three words was coded into positive and negative themes. A range of words were used and accordingly classified into ‘positive’, ‘indeterminate’ and ‘negative’. Words such as ‘exciting’, ‘rewarding’ and ‘fulfilling’ were classified as positive; ‘challenging’ and ‘steep learning curve’ were indeterminate; and ‘hard’, ‘exhausting’, and ‘chaotic’ were classified as ‘negative’. Three-word responses were collectively grouped in one of three categories. Percentages were calculated to explore trends for each time period (refer Figs. 1 and 2).

Three written words from the participants across both periods of stability and instability were the most striking difference, that is, the most significant difference was the trends in the results across the two distinct periods. Responses from graduate nurses across both unit-based and ‘mobile’ graduate nurse programs indicated relatively positive experiences during stability, especially in the ‘mobile’ program. There was a stark contrast during disruption where the feeling about the reported experiences notably changed; the graduate nurses in unit-based programs when compared with graduate nurses participating in the ‘mobile’ program, indicated more positive responses. Interestingly, at the time of the second survey (2020–2022), during the pandemic, there were still many positive statements, however also a significant number of negative statements, mostly from ‘mobile’ staff, indicating the known difficult elements. Many graduate nurses left (refer Table 2). The nurses that remained made negative comments.

#### Open-ended question - what was the impact of COVID-19 for you?

Insight as to the three-word descriptor can, in part, be provided by the responses to the open-ended question about impact of COVID-19. Content analysis of these statements identified four themes, as depicted in Table 3.

**Table 3:** “What was the impact of COVID-19 for you?”: percentage (and number) of respondents’ feedback aligning with each themed content area

Themes of feedback	Mobile graduate nurse responses	Unit-based graduate nurse responses
Education	30% (12)	25% (13)
Support	43% (17)	8% (4)
Role	65% (26)	12% (6)
Staffing	20% (8)	35% (18)
Not affected	5% (2)	15% (8)

The majority of respondents from the ‘mobile’ graduate nurse cohort commented that they did not perceive their work to be ‘nursing’. Surveillance, screening and testing, and emergent work performed by ‘mobile’ graduate nurses including very important public health measures was deemed as ‘not real nursing’ by these nurses. Nearly half, 43%, of the respondents from the ‘mobile’ graduate nurse cohort indicated they did not feel supported to conduct this role (refer Table 3). Not surprisingly, ‘mobile’ newly graduated nurses appeared most challenged by what was required of them in their role. These ‘mobile’ nurses were routinely called upon to engage with the requisite public health measures that were, at times, planned in haste and with minimal preparation for managers and staff. It is observed the graduate nurses in stable and consistent work settings who were not required to manage public health issues, while highlighting that staffing constraints impacted their graduate experience, did not identify the pandemic as affecting their role as significantly as the ‘mobile’ cohort. Across both programs nurses indicated a desire for more education.

## Discussion

This study explored two distinct support programs for newly graduated nurses commencing at a major tertiary facility, at two distinct periods, one of stability and another of instability created by the pandemic. The intent of the two graduate nurse programs is quite unique, with the unit-based program developing specialised skills, and the ‘mobile’ program focused on agility and responsiveness in unpredictable circumstances. Early in their career newly graduated nurses benefit from educational guidance and organisational support, especially assistance with confidence and social intelligence that advances adaptation [1, 24]. Organisational support programs on offer to both unit-based, and ‘mobile’ graduate nurses sought to address known challenges to assist professional, social and organisational assimilation [4, 23] through guidance to integrate into the team, coaching, and provision of regular, timely feedback.

With an overall mean retention rate between 78 and 96% across both graduate nurse programs over the period of instability, these figures are much higher than what is often reported in the literature. Of interest, retention rates two years following the initial outset of the pandemic 2021–2022, and later during the period of instability were also predominantly above international averages. The greatest attrition was of ‘mobile’ graduate nurses (50%) that commenced in 2020, during the height of uncertainty. This sub-group of the workforce employed during the period of instability was the most burdened with immediate responses that emerged with the onset of the pandemic. Employed to be responsive and fill ‘emergent’ gaps in nursing staffing numbers, often tasks were repetitious, not closely aligned to what graduate nurses understood to be professional practice, and with limited opportunities to learn what they expected nursing work to entail. Potentially, these ‘unexpected’ situations may have caused stress and, or dissatisfaction leading to high attrition. The remainder retention rates were satisfactory (75-90%), and relatively consistent despite the pandemic.

Exploration of respondent words are potentially valuable as they offer nuanced insights into retention figures. Survey results during the period of stability of the three-word response are immensely positive; especially for graduate nurses participating in the ‘mobile’ program. The alternate approach of a ‘mobile’ graduate nurse workforce offered varied experiences to increase confidence across contexts of shifting priorities. Opportunities during periods of stability demonstrated merit, possibly as this ‘mobile’ program offered diversity in conjunction with managing the ‘shock’ element. The response of graduate nurses commencing during periods of turmoil (2020–2022) of ‘mobile’ graduate nurses was in stark contrast, namely, mainly negative. The poor retention rate, coupled with discouraging sentiments evident in the survey could be attributed to high variability, poor sense of purpose, inadequate guidance, and general bewilderment: factors working against social adaptation [1, 24]. These themed factors provide a clear rationale for improving guidance, support, and clarity during graduate transition. The pandemic presented inordinate challenges, arguably ‘shock’ that was beyond what could be successfully controlled within the program. ‘Mobile’ newly graduated nurses were required to continually pivot to public health policy requirements, that they did not see relevant to their role. Ideally, more effort could have been made to reduce ‘shock’ and dissatisfaction through counselling about the value of nursing in the maintenance of public health measures and therefore the significant contribution of screening, testing and general surveillance roles. Alternatively, unit-based newly graduated nurses did not experience the same turmoil. Conceivably, unit-based graduate programs during the pandemic offered clear structure and stability when so much of the graduate nurses’ world was unpredictable. They were not required to engage extensively in general public safety measures. Unsurprisingly, guidance to assist unit-based graduate nurses’ adaptation was more readily managed.

Many lessons can be learned about graduate nurse programs and retention through this longitudinal analysis of turbulent times. In contrast to many countries, Australia was in a unique situation to employ newly graduated nurses into units that were largely protected from external impacts. In these situations that deviated little from the standard graduate nurse support, retention rates were high. These findings provide strong evidence, and in doing so, further reinforce the value of graduate nurse program requirements such as support, stability and guidance. Of note, it was demonstrated from the retention data and survey findings that during periods of stability these needs can be creatively designed through novel graduate nurse programs for ‘mobile’ units. Given the success of the ‘mobile’ workforce when resources were available, there is value in exploring how to better prepare and handle expectations in times of crisis.

Building, maintaining, and sustaining an agile, capable workforce is at the very least arduous, especially during global crises. Of critical importance is effectively engaging, supporting, and advancing capability of graduate nurses to lead and deliver quality health care now and into the future. Exploring the contribution of alternate strategies such as generic skill acquisition across multiple settings, staggered immersion across different teams, and focus on managing priorities in diverse circumstances, rather than focus on specialised capability for a designated patient cohort is paramount for safe quality care provision in versatile conditions and situations. Shifting the traditional perception of graduate nurse programs from specific task competency and organisational processes to a more considered and supported diverse approach to adaptation is imperative for addressing these healthcare service demands. Our findings suggest there is value in commissioning and providing organisational support programs for graduate nurses to create this agile and responsive workforce. In doing so, there are limitations; while the ‘unexpected’ may assist to sustain interest, it requires effective supervision, support, and clear parameters.

## Conclusions

Structured organisational and learning support can, when appropriately tailored and structured, meet the needs of newly graduated nurses so that they feel supported and competent to deliver quality care. Support and development in a nurturing environment contribute to retention of staff, regardless of the program. Considered planning of the constituent elements of support include adequate preparation of expectations and addressing what is needed to optimise adaptation. Future workforce planning must consider management of sensitivities of newly graduated nurses and furthermore, consider programs that shift the focus of opportunities and development from the dominant perceptions of nursing practice. The nursing profession needs to be prepared for future crisis. Taking risks through adjusting and adapting development and support for newly graduated nurses through alternative structured guidance is an important endeavour to build an agile workforce that can meet challenging circumstances across health care contexts now and into the future.

## Electronic supplementary material

Below is the link to the electronic supplementary material.

Supplementary Material 1

## Data Availability

The datasets used and analysed during the study are included in the manuscript and supplementary information files (Appendix A).
