# Supplementary material for: Comparative analysis of newly graduated nurse support through periods of turmoil: lessons learnt for building a future workforce for uncertain times
Source: BMC Nurs. 2024 Oct 30;23:796. doi: 10.1186/s12912-024-02460-4 (PMC11523871; doi:10.1186/s12912-024-02460-4)
Supplement: Supplementary file 1 — Supplementary Material 1 [file 12912_2024_2460_MOESM1_ESM.docx]

**Appendix A**

Graduate nurse experiences Survey Tool:

1. When did you complete your graduate year?
2. What Graduate Program did you complete [‘unit based’, or ‘mobile’]?
3. In what department did you commence your graduate year?
4. Do you still work in that unit? If not, where are you working now?
5. What is your Substantive Position?
6. How would you describe your graduate year in three words?

Additional question in the latter time period that asked about impact of COVID-19:

1. What was the impact of COVID-19 for you?
